# A cheminformatics and network pharmacology approach to elucidate the mechanism of action of *Mycobacterium tuberculosis* γ-carbonic anhydrase inhibitors

**DOI:** 10.3389/fphar.2024.1457012

**Published:** 2024-09-02

**Authors:** Ajay Manaithiya, Ratul Bhowmik, Kunal Bhattacharya, Rajarshi Ray, Sagar Singh Shyamal, Fabrizio Carta, Claudiu T. Supuran, Seppo Parkkila, Ashok Aspatwar

**Affiliations:** ^1^ Faculty of Medicine and Health Technology, Tampere University, Tampere, Finland; ^2^ Pratiksha Institute of Pharmaceutical Sciences, Guwahati, Assam, India; ^3^ Royal School of Pharmacy, The Assam Royal Global University, Guwahati, Assam, India; ^4^ Department of Pharmaceutical Engineering and Technology, Indian Institute of Technology (Banaras Hindu University), Varanasi, India; ^5^ Department of Neuroscience, Psychology, Drug Research, and Child’s Health, Section of Pharmaceutical and Nutraceutical Sciences, University of Florence, Florence, Italy; ^6^ Fimlab Ltd., Tampere University Hospital, Tampere, Finland

**Keywords:** *Mycobacterium tuberculosis*, γ-CAs, virtual screening, system biology, inflammatory responses

## Abstract

**Background:**

*Mycobacterium tuberculosis* (Mtb) carbonic anhydrases (CAs) are critical enzymes that regulate pH by converting CO_2_ to HCO_3_^−^, essential for Mtb’s survival in acidic environments. Inhibiting γ-CAs presents a potential target for novel antituberculosis drugs with unique mechanisms of action.

**Objective:**

This study aimed to explore the biological connections underlying Mtb pathogenesis and investigate the mechanistic actions of antituberculosis compounds targeting the Cas9 protein.

**Methods:**

We employed homology modeling and virtual screening to identify compounds with high binding affinities for Cas9 protein. This study used the homology modeling approach employing high-quality AlphaFold DB models for γ-CA. Furthermore, the systems biology approach was used for analyzing the integrated modelling of compounds, integrating data on genes, pathways, phenotypes, and molecular descriptors. Single-cell RNA sequencing was also conducted to profile gene expression.

**Results:**

Three compounds, F10921405, F08060425, and F14437079, potentially binding to Cas9 protein, have been identified. F10921405 and F08060425 showed significant overlap in their effects on pathways related to the immune response, while F14437079 displayed distinct mechanistic pathways. Expression profiling revealed high levels of genes such as PDE4D, ROCK2, ITK, MAPK10, and SYK in response to F1092–1405 and F0806-0425, and MMP2 and CALCRL in response to F1443-7079. These genes, which play a role in immune modulation and lung tissue integrity, are essential to fight against Mtb.

**Conclusion:**

The molecular relationship and pathways linked to the mentioned compounds give the study a holistic perspective of targeting Mtb, which is essential in designing specific therapeutic approaches. Subsequent research will involve experimental validation to demonstrate the efficacy of the promising candidates in Mtb infections.

## Graphical Abstract

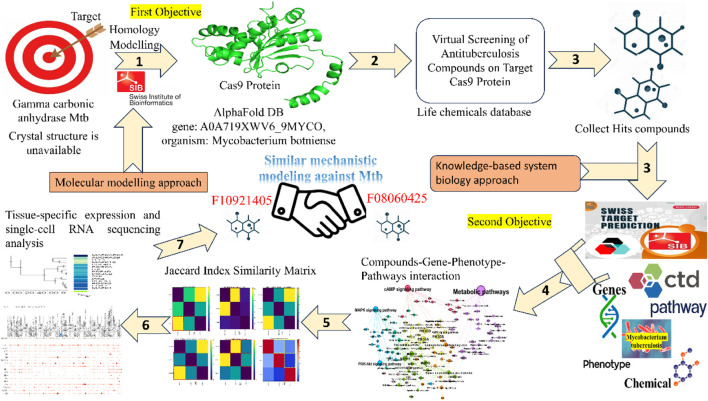

## Introduction

*Mycobacterium tuberculosis* (Mtb), the causative agent of tuberculosis (TB), is an extremely infectious pathogen that is well adapted to survive within the host without causing the disease (World Health Organization, 2023; Bhowmik et al., 2023). In the latent phase of infection, Mtb uses a range of effector proteins to evade the host immune system, adopts itself, and resides in granulomas, the highly developed and organized structures of the host, in response to persistent infection. TB is one of the oldest recorded human diseases and still one of the biggest killers among infectious diseases despite the availability of the BCG vaccine and several lines of antibiotics (World Health Organization, 2023; Dos Santos et al., 2024; Parkkinen et al., 2024). Worldwide, 10.6 million people developed active TB, causing 1.3 million deaths (World Health Organization, 2023). In addition, an estimated 23% of the global population is infected with latent TB (Houben and Dodd, 2016). The situation is aggravated by the emergence of drug-resistant Mtb strains due to indiscriminate use of antibiotics and non-adherence to the treatment regime. Although both drug-susceptible and drug-resistant TB is treatable (World Health Organization, 2023; Aspatwar et al., 2019a) using currently used antibiotics that include second-line and third-line anti-TB drugs (Aspatwar et al., 2019a). However, these drugs are extremely toxic, expensive, and consist of lengthy treatment regimes, making them unsuitable for treating TB. Therefore, there is an urgent need for novel treatment methods that target yet unknown but crucial pathways and the proteins that are involved in these pathways, which are essential for the life cycle of Mtb and its virulence. The drugs shorten the treatment time, with minimal side effects, and are affordable. One of the approaches is to develop compounds that inhibit the activity of these proteins and disrupt the novel pathways of the bacteria. To combat this successful human pathogen, we need a better understanding of the basic biology of mycobacterial pathogenesis and identify the proteins that play a pivotal role in the survival and pathogenesis of Mtb in the host. The sequencing of the mycobacterial genome has identified several proteins that are essential for the bacterium (Aspatwar et al., 2019a; Aspatwar et al., 2018a; Aspatwar et al., 2017). Among these, the genome of Mtb encodes for three β-carbonic anhydrases and one γ-carbonic anhydrase. This understanding is not only crucial for the development of novel compounds but also for the advancement of our knowledge in the field of microbiology and infectious diseases.

Carbonic Anhydrases (CA EC 4.2.1.1) catalyze a simple yet physiologically significant enzymatic reaction in all living organisms, i.e., reversible hydration of CO_2_ to HCO_3_^−^ and H^+^ (Aspatwar et al., 2022a). In addition, it has been demonstrated that in living organisms, the CAs play several physiological roles, namely, respiration, pH homeostasis, CO2 sensing, and ion transport (Aspatwar et al., 2023a; Aspatwar et al., 2018b; Aspatwar et al., 2010; Aspatwar et al., 2019b). In Mtb, β-CAs are involved in survival during starvation, hypoxia, stress conditions in the host granuloma, biofilm formation, virulence, and extracellular DNA transport. Several *in vitro* and *in vivo* studies demonstrated that β-CAs of Mtb can be targeted using small molecular inhibitors (Aspatwar et al., 2019a; Aspatwar et al., 2017; Abdoli et al., 2023; Aspatwar et al., 2023b; Aspatwar et al., 2022b; Aspatwar et al., 2020). The database search sequence analysis showed that Mtb γ-carbonic anhydrases (γ-CAs) is an enzymatically active protein playing a crucial role in maintaining the bacterium’s intracellular pH balance and facilitating its metabolic functions. This enzyme’s activity is vital for the survival and pathogenicity of Mtb within the host. However, a crystal structure for γ-CA-Mtb is currently unavailable, pointing to a significant gap in structural knowledge. Targeting γ-CA represents a promising therapeutic strategy since its inhibition could disrupt these critical biological processes, potentially reducing bacterial virulence and offering effective treatment for tuberculosis.

Over the past decade, numerous studies have focused on developing effective *in vitro* carbonic anhydrase inhibitors (CAIs) aimed at pathogenic enzymes, with the goal of translating these results into *in vivo* applications and eventually clinical settings. Despite this progress, CAIs have not yet gained serious consideration as anti-infectives, largely because dedicated drug discovery programs are lacking. However, some compounds, such as selenazoles with benzenesulfonamide derivatives and other CA inhibitors, show potential in combating drug-resistant microbes, including bacteria, fungi, and protozoa (Angeli et al., 2019; Aspatwar et al., 2019c). Recently, γ-CAs have emerged as promising antibacterial targets, with potential applications in treating diseases like melioidosis (Di Fiore et al., 2022; Supuran, 2011). Furthermore, inhibition studies of various clinically relevant CAIs, such as acetazolamide, benzolamide, and brinzolamide, reveal a spectrum of activity across Beta carbonic anhydrases (β-CA1, β-CA2, and β-CA3). In contrast, inhibitors like saccharin and sulpiride show higher KIs, indicating less selectivity and efficacy (Davis et al., 2011; Nishimori et al., 2006; Carta et al., 2009; Ceruso et al., 2014). Acetazolamide, for instance, demonstrates potent inhibition of β-CA2 with a KI of 9.8 nM, indicating its strong inhibitory capacity (Ommi et al., 2023). Newer classes of CAIs, including dithiocarbamates and pyrazole-tethered sulfamoylphenyl acetamides, also show nanomolar-range inhibition constants, particularly against β-CA3 (Aspatwar et al., 2020; Ommi et al., 2023). These findings underscore the therapeutic potential of CAIs and highlight the need for further exploration of CA inhibitors as viable strategies for targeting γ-CA in *mycobacterium tuberculosis*. The promising results seen *in vitro* suggest that a focused drug discovery effort could yield novel therapeutics for combatting a range of infectious diseases, especially those caused by drug-resistant pathogens.

The γ-CAs are widely distributed in diverse species from all three domains of life. The first γ-CA was reported from Methanosarcina thermophila, an anaerobic methane-producing species from the Archaea domain. Further characterization showed that this CA is catalytically active and is iron-dependent *in vivo* (Alber and Ferry, 1994; Ferry, 2010; Macauley et al., 2009). The γ-CA from the Archaea domain is a homotrimer, and the crystal structure reveals monomers with a distinctive left-handed parallel β-helix fold (De Luca et al., 2016). In γ-CAs, zinc ion is coordinated by three histidine residues and a water molecule/hydroxide ion like α-CAs.

Histidine residues from the monomers ligate with three active site metals surrounded by residues in a hydrogen bond network essential for activity. Although there is no similarity between active residues of γ-CA and other CA families, kinetic analyses show a two-step mechanism analogous to the other CAs investigated. Subsequently, γ-CAs were found to be widely distributed in bacteria and the mitochondria of plants (Angeli et al., 2018; Del Prete et al., 2014; De Luca et al., 2015). In pathogenic bacteria, the γ-CAs have been shown to be novel antibacterial drug targets (Supuran and Capasso, 2018). These findings are important for developing novel antituberculosis drugs targeting γ-CA of *Mycobacterium tuberculosis*. We are designing a pipeline on Mtb CA targets where mechanistic interpretation of molecular and chemical signatures can be explored, and some results are undergoing the peer review process. In this work, the gamma (γ)-CA could be a novel target of the Mtb for developing drugs that are effective in treating TB and devoid of resistance by the bacterium. Among the CAs from pathogenic bacteria, gamma-CAs have been the enzymes that have been the least investigated. With a research project on developing anti-TB drugs targeting gamma-CAs of Mtb, we employed homology modeling and virtual screening to identify top compounds with a potent binding affinity for Cas9 in-depth study. We also had a limited number of high-confidence AlphaFold DB models that we used for homology modeling, which was consistent with the accuracy and reliability of our results. After that, we explored the biological networks of biological interactions among relevant genes associated with Mtb pathogenesis and their involvement in specific biological pathways and phenotypes. Moreover, graphical networks and mathematical correlations revealed biologically and mechanistically relevant profiles linking Mtb pathogenesis with the target compounds (Table 1). This comprehensive approach may also elucidate gene expression within lung tissues, providing a more profound understanding of the underlying interactions.

**TABLE 1:** Tabular representation of computational tools and algorithms for mechanistic bioinformatics Analysis.

Tools/Methods used	Background of methodology	Key function and motive
AlphaFold DB	Utilizes deep learning to predict protein structures from amino acid sequences	To predict the 3D structure of proteins for further biochemical analysis
SWISS-MODEL	Automated homology-modeling server using comparative modeling to build 3D protein models	For creating 3D models of protein structures when experimental data are unavailable
PyRx 0.8 and Auto Dock	PyRx is a virtual screening software that uses AutoDock for docking. AutoDock is a suite of automated docking tools designed to predict how small molecules bind to a receptor of known 3D structure. Utilizes a genetic algorithm (GA) for docking simulations	To screen large libraries of compounds and predict their binding affinities to protein targets
Comparative Toxicogenomics Database (CTD)	Integrates data from literature to illuminate gene, chemical, and disease interactions	To obtain biological data relevant to diseases or biological conditions
Venny 2.1.0	Interactive tool using set theory to show relationships with Venn diagrams	To identify overlapping or unique genes across multiple biological conditions
Correlation analysis for cluster determination	Uses statistical measures like the Jaccard Index and similarity matrices to evaluate the degree of overlap or similarity between data sets, which can include gene expression profiles or other molecular data $J\;\left(A,B\right)=\frac{\left|A\cap B\right|}{\left|A\cup B\right|}$ where A and B are sets of elements (such as gene expression profiles or molecular data), J (Jaccard Index). J = 0 (completely dissimilar), 1(completely similar)	To identify clusters of genes that share similar expression patterns or functions
GTEx portal	A public resource that provides tissue-specific gene expression and regulation data and collected from a variety of tissues	To explore organ-specific and single-cell gene expression data to understand gene regulation
Cytoscape 3.10.2/Network X/Gephi 0.10.1	These tools are used for visualizing and analyzing network data (Graph-based modeling and node-edge interactions). Directed graph method was used for analysis	To visualize and analyze complex biological networks, understanding interactions at the molecular level

## Experimental section

### Homology modelling

The homology model for Gamma carbonic anhydrase from *Mycobacterium tuberculosis* (Gamma CA-Mtb) is currently unavailable. Consequently, we selected a closely related AlphaFold DB model of Gamma carbonic anhydrase from *Mycobacterium* botniense (gene: A0A719XWV6_9MYCO) based on its high sequence identity and structural coverage. So, the Cas9 protein sequence was obtained in FASTA format and used as the target for homology modeling. The template for the homology modeling was selected based on sequence similarity and structural coverage. The chosen template was the AlphaFold DB (https://alphafold.com/) model of A0A719XWV6_9MYCO (gene: A0A719XWV6_9MYCO, organism: *Mycobacterium* botniense), representing a gamma carbonic anhydrase family protein. This model exhibited a GMQE score of 0.98 and had an identity score of 85.63, indicating a high-quality template for our modeling purposes. The homology modeling was performed using SWISS-MODEL (https://swissmodel.expasy.org/) leveraging the AlphaFold v2 method to predict the tertiary structure of the Cas9 protein.

The SWISS-MODEL alignment tools generated the alignment between the Cas9 protein sequence and the template. Special attention was paid to aligning conserved regions and active sites to ensure functional integrity in the model. The 3D model of Cas9 was then constructed based on the alignment, with the software generating loops and side-chain conformations in regions where the target and template differed.

### Virtual screening of antituberculosis compounds against Cas9

#### Compound library acquisition

For the virtual screening, we focused on identifying potential inhibitors of the Cas9 protein with antituberculosis activity. To this end, we procured targeted and focused antituberculosis screening libraries containing drug-like compounds. These libraries were curated based on fingerprint similarity and molecular docking studies for the InhA enzyme, an essential component in *Mycobacterium tuberculosis*. The chemical libraries were downloaded in Structure Data File (SDF) formats from the Life Chemicals database (https://lifechemicals.com), a comprehensive repository of small molecules designed for drug discovery projects.

### Compound library filtration and selection

#### Library composition and pre-filtering

The initial compound libraries comprised a collection of 4,254 compounds in the antituberculosis-focused library and 7,464 compounds in the targeted library. These libraries were assembled to identify potential tuberculosis inhibitors, specifically curated to include compounds with structural features and properties relevant to the disease’s molecular targets.

## Application of drug-likeness and physicochemical filters

We applied a series of drug-likeness and physicochemical filters to refine the libraries based on established criteria. The Lipinski’s Rule of Five, Veber’s Rules, and Ghose’s Filter were employed as the primary filters to identify compounds with favorable oral bioavailability and pharmacokinetic properties. This rigorous filtering process reduced the focused library to 170 compounds and the targeted library to 57 compounds, ensuring that the remaining molecules possessed drug-like attributes suitable for further evaluation.

### Toxicophore screening

We then subjected these selected compounds to toxicophore screening to identify any structural features associated with toxicity, thereby enhancing the safety profile of the lead candidates. The toxicophore analysis was performed using an *in silico* approach, efficiently pinpointing potential toxicity risks. As a result, 148 compounds from the focused library and 48 from the targeted library successfully passed the toxicophore screening, indicating a minimized likelihood of adverse toxicological effects.

### Pan-assay interference compounds (PAINS) filtration

Further refinement was achieved by filtering out Pan-Assay Interference Compounds (PAINS). PAINS are chemical compounds that are frequently seen as false positives in high-throughput screenings, which can lead to misleading conclusions about a compound’s bioactivity. After applying the PAINS filter, the focused library was narrowed down to 140 compounds, while the targeted library maintained 48 compounds.

### Final compound selection for virtual screening

The culmination of these sequential screening steps resulted in a total of 188 compounds deemed suitable for virtual screening against the Cas9 protein. This carefully curated set of compounds merged both the filtered focused and targeted libraries, representing the most promising candidates for further computational and biological analyses.

## Virtual screening against Cas9

### Preparation of ligand library

The 188 molecules emerging from toxicophore screening and PAINS analysis were prepared for molecular docking. Utilizing the Universal Force Field (UFF), the compounds were energy-minimized to establish stable, low-energy conformations. This step was imperative to ensure that the ligands’ geometries were optimized for accurate interaction with the Cas9 protein binding site.

### Molecular docking protocol

Molecular docking was performed using PyRx 0.8 (Dallakyan and Olson, 2015), which utilizes AutoDock Vina (Trott and Olson, 2010) for docking simulations. This virtual screening was conducted against the homology model of Cas9, with the binding affinity of each ligand calculated to predict interaction strength and stability. Docking parameters were set to adequately cover the whole protein and enhance the search for optimal binding modes. The grid box for the docking was defined with the following parameters: center_x = −1.7521, center_y = −0.3371, center_z = −0.0579, size_x = 41.9948, size_y = 41.1091, size_z = 45.2622 (units in Ångströms), with an exhaustiveness of eight to ensure thorough sampling of the conformational space.

### Data sources and retrieval for system biology analysis

We employed the final screened molecules from molecular docking for further systems biology analysis. For target prediction, three molecules, F10921405, F08060425, and F14437079, were analyzed using the “Swiss Target Prediction” server (http://www.swisstargetprediction.ch/). We identified 100, 56, and 10 targets for these molecules, respectively (Supplementary Material). To obtain detailed mechanistic information associated with *mycobacterium tuberculosis* (Mtb), we utilized the “Comparative Toxicogenomic Database” (https://ctdbase.org/). This database integrates extensive information regarding genes, phenotypes, and Pathway associated with Mtb. We hoped to achieve a comprehensive understanding of the dynamics and interplay amongst different levels of observed interactions during *mycobacterium tuberculosis* by integrating these data. Then we performed Venny Plot (https://bioinfogp.cnb.csic.es/tools/venny/) to get unique sets of genes, pathways, and connectivity related to disease. The set intersection method allowed us to identify overlapping genes/pathways/phenotypes among the predicted targets of the three molecules and their association with tuberculosis. The integrated approach gave unique insights into molecular mechanistic and therapeutic targets to combat Mtb.

### Disease associated with compounds-gene-phenotype-pathways interaction

To process and visually represent the selected compound interactions, along with disease associated with their phenotypes, genes, and pathways, we utilized Python libraries such as Pandas (https://pandas.pydata.org/), NetworkX (https://networkx.org/), and Matplotlib (https://matplotlib.org/) to handle the dataset. Apart from, we also used Gephi (https://gephi.org/) and Cytoscape (https://cytoscape.org/) for visualisation of biological signatures interaction. The data was filtered through filtering to concentrate on specific interest entities. The data subset was used to construct biological networks that consisted of the nodes corresponding compounds, genes, phenotypes, and pathways. Edges between nodes were generated based on direct graph that showed as biological connection to each other.

We used Matplotlib to create a color-coded visualization of these graphs to better understand the nodes associated with each in the form of compound, gene, phenotype, or pathway by marking them with distinct colours. Since spacing the nodes effectively made the graph clearer, we decided to use a degree layout in NetworkX and Cytoscape. We also analyzed the frequency of associated pathways, phenotypes, and genes for each compound. In order to assess how the biological features are associated across our data set, a frequency distribution analysis was developed using Python Panda’s library. Finally, to calculate the frequency of occurrence in the dataset for a specific category c (where c can be genes, pathways, or phenotypes) an equation is used:

$$f\left(c\right)=\frac{n\left(c\right)}{N}$$

The number of occurrences of a category, whereas n(c) refers to the number of occurrences of a specific category c (e.g., gene, Pathway, or phenotype), and N is the total number of records analyzed.

### Gene frequency matrix for phenotype-pathway associations and similarity analysis

We sought to determine the classes with the highest gene involvement by calculating the frequency of genes across each Pathway and phenotype. This approach allowed us to identify the phenotypes and pathways most associated with the genes and compounds involved in the pathogenesis of Mtb. We selected classes containing more than 10 genes and used similarity matrix calculations for gene pair to determine the occurrence of shared phenotypes and pathways. We built the similarity matrix (SM). The SM results from the number of common elements in pairs between analyzed samples divided by the total element number in each pair of samples, equal to a value from 0 to 1. Higher values of SM mean a higher concentration of shared biological features. Heatmaps index were generated from the SM matrix to show the results visually. Darker clusters in the heatmaps index represent the magnitude of gene pair relationships, and deeper colored clusters represent stronger calculated pairs. From this heatmap index analysis, we pinpointed those genes and molecular players that are most involved in the pathogenic reaction associated with Mtb progression.

### Tissue-specific expression and single-cell RNA sequencing analysis

The significance of tissue-specific expression and single-cell RNA sequencing analysis were performed by the GTEx portal (https://www.gtexportal.org/home/). We filtered out the tissue-specific organ, focusing on lung tissue for further analysis. The analysis involved determining the percentage of detected cells in the lungs and their involvement. In tissue-specific expression, we utilized clustering to identify genes’ common modes of action, categorizing them into high or low expression within lung tissue. Based on single-cell RNA sequencing analysis, these clusters highlight the complex interplay between different lung cell types in responding to *mycobacterium tuberculosis* (Mtb) infection. This approach allows us to understand the differential gene expression patterns and their potential roles in the pathogenesis and immune response against Mtb.

## Results and discussion

Initially first objective, we performed homology modeling of the Cas9 protein sequence using the high-quality AlphaFold DB model of A0A719XWV6_9MYCO as the template, achieving strong alignment, particularly in conserved regions and active sites. Following this, virtual screening was initiated using targeted and focused antituberculosis compound libraries from the Life Chemicals database. After applying drug-likeness and physicochemical filters, toxicophore screening, and filtering out Pan-Assay Interference Compounds (PAINS), we refined our selection to 188 compounds. These were prepared and energy-minimized for molecular docking using PyRx tool with AutoDock Vina, targeting the homology model of Cas9 to predict binding affinities and interaction stabilities (Supplementary Table S1). At the end of the screening process, we identified the top three hit molecules, which exhibited the highest binding affinities and potential as antituberculosis agents targeting the Cas9 protein (Supplementary Table S2).

Furthermore, moving on to the second objective, we developed a computational framework to investigate the complex molecular mechanisms of *mycobacterium tuberculosis*. Our approach integrates multiple data dimensions to deepen our integrated modelling understanding of tuberculosis disease: disease-associated genes, disease-associated phenotypes, disease-associated pathways, compounds targeting disease-associated genes, and prediction of substructure fingerprint descriptors. Combining a “Comparative Toxicogenomic Database” such as phenotypes and compounds with detailed mechanistic information, including genes and pathways, provides a comprehensive view of the interactions and processes driving tuberculosis. This model helps us understand more about the disease from a biological perspective and creates new opportunities for developing treatments targeting specific areas. Specifically, our dataset comprises information on 34,889 disease-associated genes, 72,676 pathways and phenotypes linked to disease states, and 146 compounds targeting diseases-related genes (Supplementary Tables S3–S5). The volume and diversity of data available for these dimensions vary significantly, reflecting the complexity of disease mechanisms captured in our study.

## Binding affinities and ligand interactions

The virtual screening of the initial 188 compounds led to identifying three lead compounds with notable binding affinities to the Cas9 protein. Compound F1092-1405 showed a binding affinity of −8.6 kcal/mol, Compound F0806-0425 displayed a slightly higher affinity of −8.7 kcal/mol, and Compound F1443-7079 demonstrated the strongest interaction with a binding affinity of −9.4 kcal/mol. These binding affinities suggest a substantial likelihood of these compounds acting as effective inhibitors of the Cas9 protein.

**FIGURE 1:**
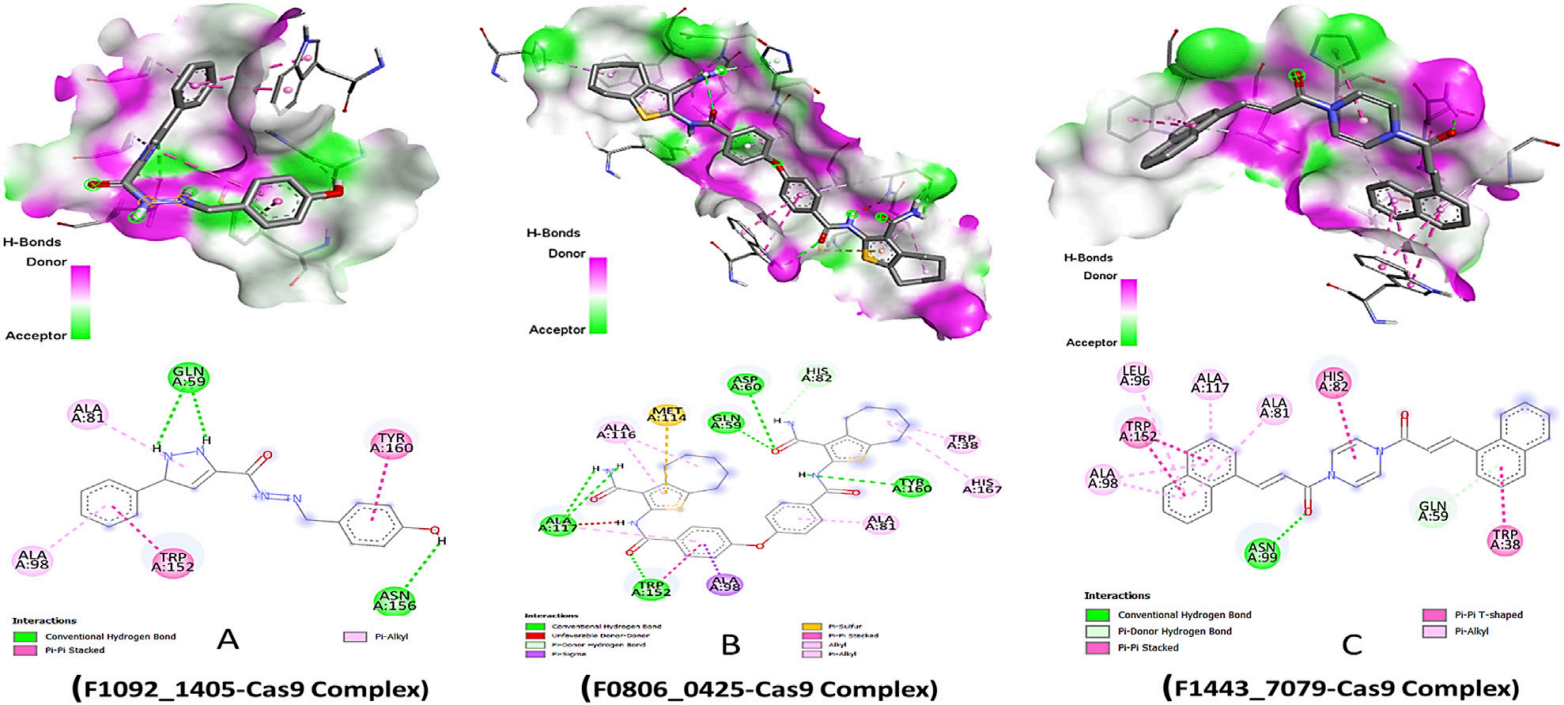
Molecular Docking 3D and 2D Interactions of Ligands with the Cas9 Protein. Each image number, A-C, showed respective compound complex.

The lead compounds demonstrated distinct modes of interaction with the Cas9 protein, which indicated their inhibitory potential. Compound F1092-1405 displayed a binding affinity of −8.6 kcal/mol, forming conventional hydrogen bonds with the amino acid residues Gln A:59 and Asn A:156. These hydrogen bonds are critical for the stability of the ligand-protein complex. Additionally, pi-alkyl and Pi-Pi stacked interactions were observed with Ala A:81, Ala A:98, Trp A:152, and Tyr A:160, respectively, suggesting a hydrophobic interaction landscape that contributes to the compound’s binding within the active site of Cas9 (Figure 1).

Compound F0806-0425, with a binding affinity of −8.7 kcal/mol, demonstrated a complex network of interactions that secured its position within the Cas9 binding site. This compound formed conventional hydrogen bonds with Asp A:60, Gln A:59, Ala A:117, and Tyr A:160, establishing a strong electrostatic linkage to the protein. Moreover, a pi-sulfur interaction with Met A:114 and a pi-sigma interaction with Ala A:98 further anchored the compound, indicating a robust interaction pattern that might contribute to a potential inhibitory effect. The most potent binder, Compound F1443-7079, presented the highest binding affinity of −9.4 kcal/mol, characterized by a distinctive binding profile. It established a conventional hydrogen bond with Asn A:99, critical for the specificity of the interaction. The compound also engaged in a pi-donor hydrogen bond with Gln A:59 and pi-alkyl interactions with Ala A:98, Leu A:96, Ala A:117, and Ala A:81, revealing a propensity to form multiple non-covalent interactions. Remarkably, a pi-pi stacked interaction with Trp A:152 and a pi-T-shaped interaction with Trp A:38 suggested a highly specific binding mode, where aromatic rings are involved in electron-rich interactions, potentially increasing the binding strength and specificity (Figure 1). These pi interactions, complemented by the alkyl interactions, might be the underlying reason for the superior binding affinity of this compound, setting it apart as the most promising candidate among those screened.

### Identification of common gene

**FIGURE 2:**
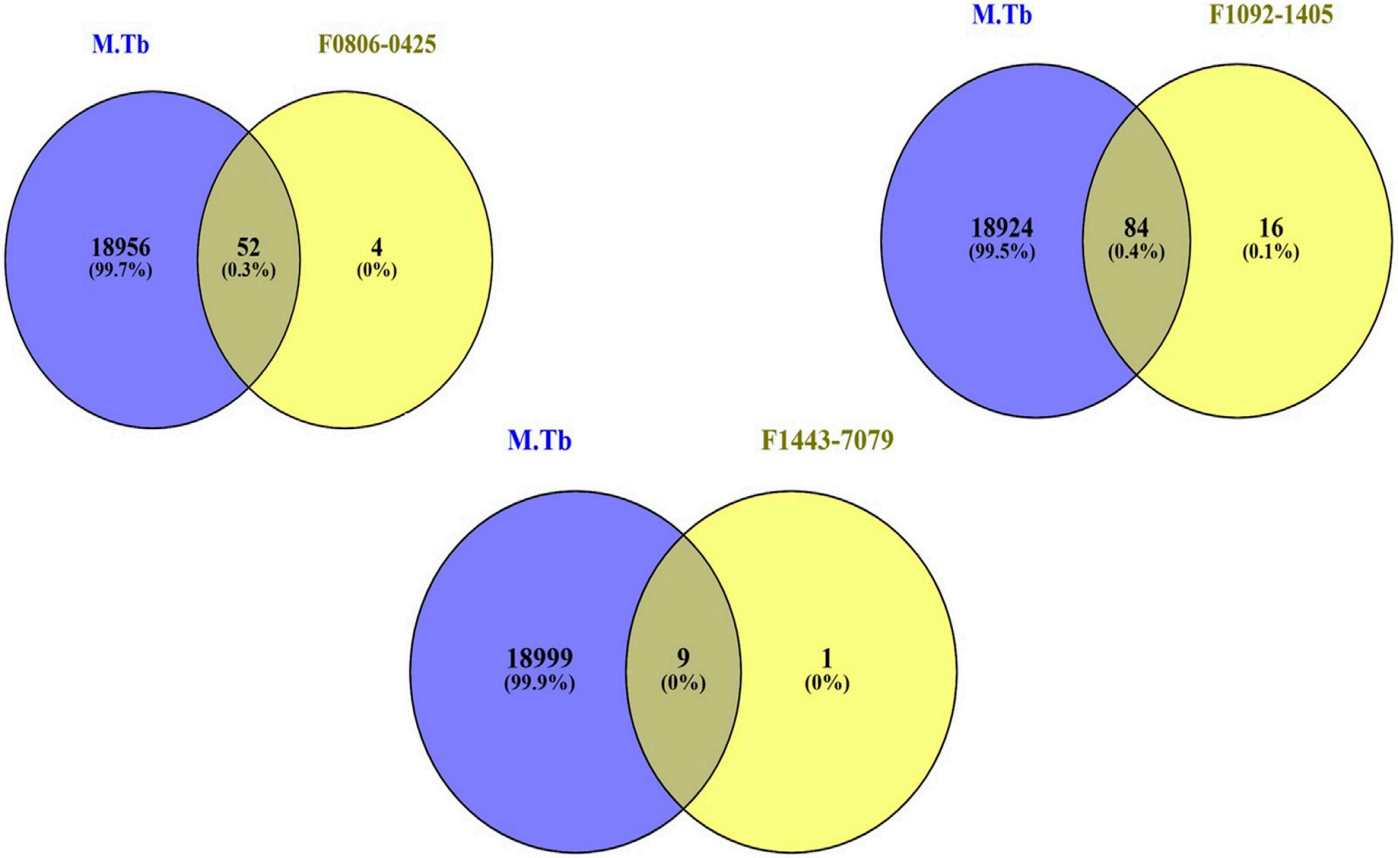
The Venn plot illustrating the common interactions between the targets of compounds and Mtb genes.

We sought to identify common genes that could serve as pharmacological targets for compounds associated with the Mtb. The Venn diagrams present common gene sets between *mycobacterium tuberculosis* (Mtb) containing genes and compounds F08060425, F10921405, and F14437079 targeting genes (Figure 2).

### Insights from interactions between gene-pathway-phenotype-compounds

From biological networks, the network diameter and radius are both one, meaning the longest possible distance between any two nodes is small. Additionally, this attribute can be highlighted by observing a characteristic path length of 1.0, implying that all the network nodes can be reached directly from any other node. In other words, there is a direct link between each pair of vertices present in this graph (Supplementary Table S6). This finding is confirmed by having 463 shortest paths among 46,300 pairs representing only one percent (or less) connection rates between them. A network’s clustering coefficient is zero, meaning no groups or clusters with internal solid connections exist. However, this does not mean the network has isolated nodes or self-loops. Furthermore, biological network analysis graph was drawn from Gephi tool to visualize graph and find out the node size of gene and pathways (Supplementary Tables S7, S8). Subsequently, we constructed biological networks with the help of also NetworkX graph where nodes represented compounds, genes, phenotypes, and pathways, connecting them if they directly interacted, such as a gene being involved in a pathway or a compound influencing a gene.

**FIGURE 3:**
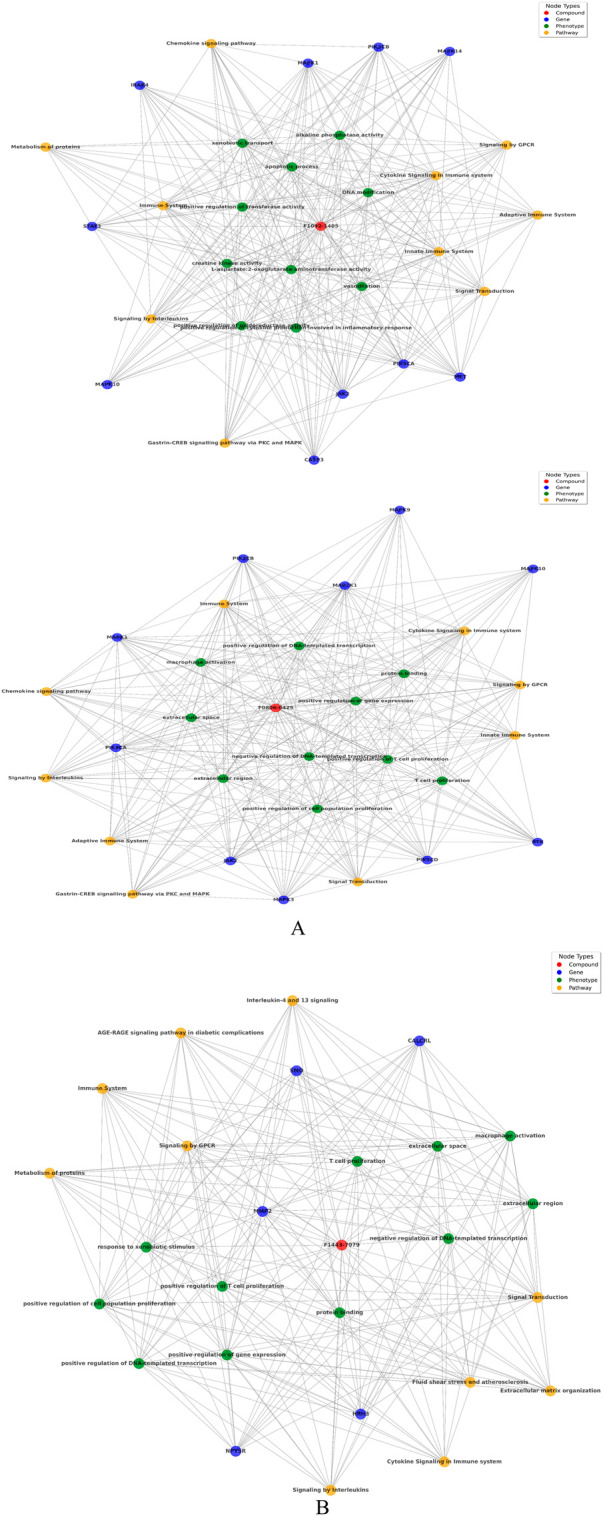
**(A)** Network analysis between compound, gene, Pathway, and phenotypes: Upper panel of image shows compound F1092-1405 and lower panel of image shows F0806-0425. **(B)** Network analysis between compound F14437079 with respect of gene, Pathway, and phenotypes.

The biological networks for compound F10921405 highlights extensive interactions with critical pathways such as Chemokine signaling, Cytokine signaling in the immune system, and the Innate immune system. The involvement of genes like MAPK1 and PIK3CA suggests that F10921405 could influence key signaling mechanisms that regulate macrophage activation and cytokine production, which are critical components in the containment and eradication of Mtb. Enhancing these pathways might help overcome the immune evasion strategies employed by Mtb, such as inhibiting phagosome-lysosome fusion in macrophages. This could potentially make F10921405 a molecule to boost immune response in TB treatment. These interactions suggest a strong involvement of compound F10921405 in modulating immune responses, potentially affecting inflammatory response. Compound F08060425 also exerts an influence on the immune system. This significantly affects key pathways as Cytokine signaling, Chemokine signaling, and the Adaptive immune system. The compound interacts with macrophage activation phenotypes and T-cell proliferation, along with the key genes FLT3, SYK, ROCK1, etc., suggesting potential involvement in immune modulation directly involving these cells (Figure 3A). Finally, compound F14437079 shows interactions with the Interleukin-4 and 13 signaling pathways, AGE-RAGE signaling, and affects phenotypes such as immune response to stimulus and regulation of gene expression. This compound also participates in interactions with cell cycle regulation genes (such as CDK2) as well as signal transduction and immune response-related factors (like the kinases MAPK9 SMO), indicating potential roles in inflammatory conditions (Figure 3B). Based on its interaction with the “Chemokine Signaling Pathway” and “Adaptive Immune System,” compound F08060425 could regulate immune responses necessary for establishing long-lasting immunity to TB. The involvement of the FLT3 gene points to possible effects on dendritic cell function and T-cell activation. Because an efficient T cell response is imperative for controlling and clearing Mtb infection, targeting this Pathway may improve the efficacy of host immunity against TB, which could support vaccine or therapeutic strategies designed to enhance cellular immune responses in TB patients (Triccas et al., 2007; Zhang et al., 2015).

Focusing on “Interleukin-4 and 13 signaling,” compound F14437079 might influence the Th2 immune response, which is generally thought to counteract the Th1 response crucial for fighting TB. However, modulating this Pathway could be useful in scenarios where a hyperactive Th1 response contributes to excessive inflammation and tissue damage, as seen in severe cases of TB (Zeng et al., 2022). Furthermore, the “AGE-RAGE signaling in diabetic complications” relevance suggests potential utility in TB patients co-morbid with diabetes, a common condition that complicates TB treatment and outcomes (Kumar et al., 2019). The presence of CDK2 and MAPK9 as central nodes involved in cell cycle regulation and signal transduction suggests that F14437079 could influence cell proliferation and immune cell function, possibly offering avenues for intervention in inflammatory diseases. The frequency distribution chart analysis follows this to find pathways, genes, and phenotypes most associated with the compounds, which offers insight into the primary biological actions or effects of these compounds. These findings are further supported by the combined similarity matrix analysis, which illustrates high similarity scores between F08060425 and F14437079 and a moderate score between compounds F10921405 (Figure 5). This implies that these compounds can be used synergistically against many facets of Mtb biology.

### Insights from similarity matrix analysis of gene-pathway-compounds-molecular descriptors

**FIGURE 4:**
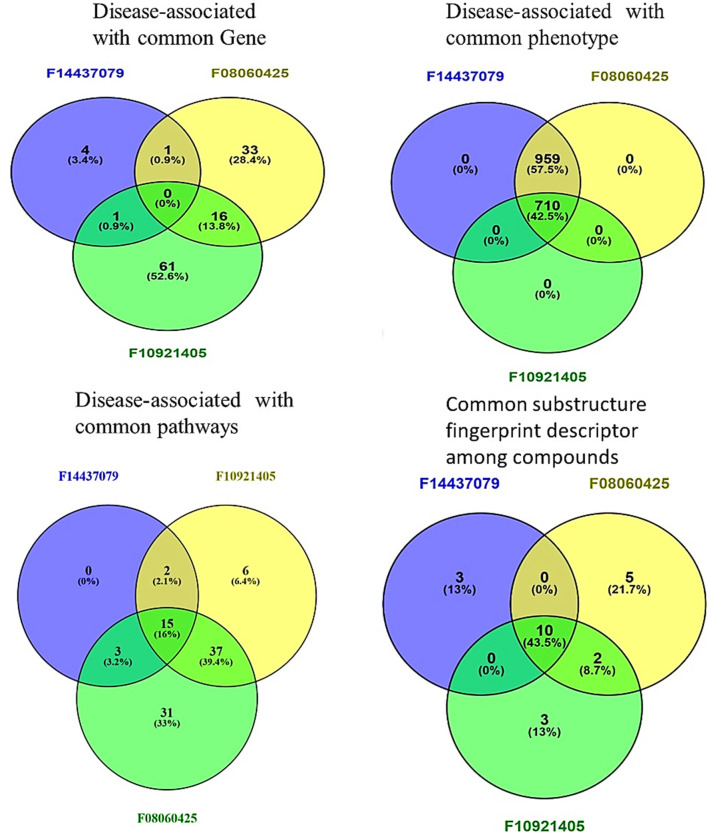
Comparative Analysis of compounds F08060425, F10921405, and F14437079 in Biological and Chemical interaction. Venn Diagrams demonstrate common interactions to each other’s between genes, phenotypes, pathways, and chemical descriptors.

**FIGURE 5:**
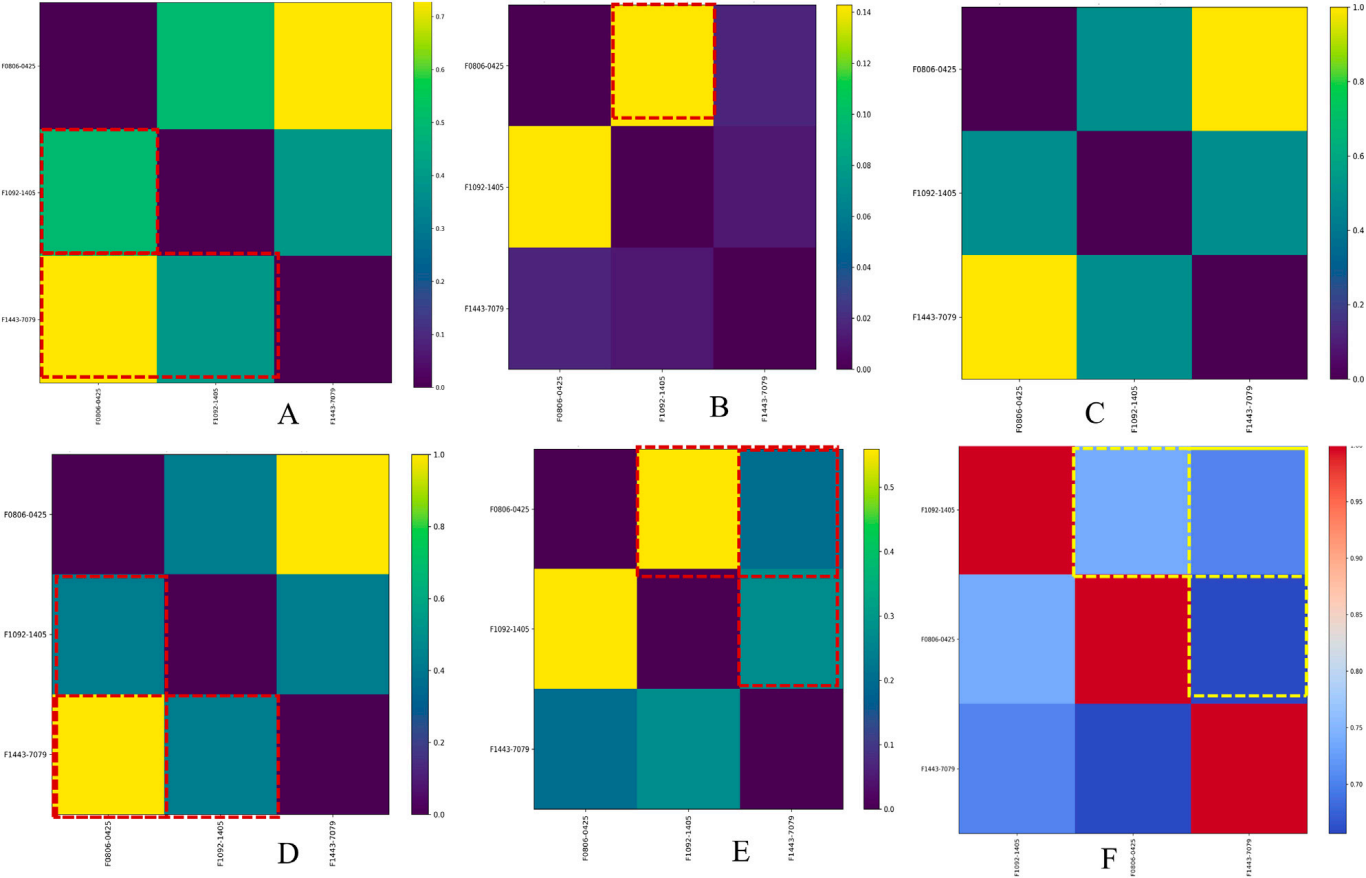
Similarity matrix for Mtb disease-associated phenotype, pathways, and genes averaged with molecular descriptors across hit compounds. Image **(A)** shows the similarity matrix between compounds, Image **(B)** shows the similarity matrix of compounds versus target genes, Image **(C)** shows the similarity matrix of compounds versus disease names, Image **(D)** shows the similarity matrix of compounds versus phenotype names, Image **(E)** shows the similarity matrix of compounds versus pathway names, and Image **(F)** shows the similarity matrix of compounds based on molecular descriptors.

The combined similarity matrix is the comparison of compounds F08060425, F10921405, and F14437079 based on various biological parameters such as genes/pathways/phenotypes/molecular descriptors set (Figure 4). Such an analysis can be important to begin to understand the potential for antimycobacterial modes of action of these compounds. The similarity score between compound F08060425 and F10921405 is 49.48%, meaning that half of analyzed biological parameters from these two compounds are the same. These data imply that F08060425 and F10921405 might share many mechanistic activities, potentially compelling the same Mtb pathogenic pathways or targets. However, the similarity score between F08060425 and F14437079 is 73.48%, suggesting a substantial overlap between these two compounds. The high similarity percent between F08060425 and F14437079 suggests that those two compounds may have the similar biological process and might have similar therapeutic effects against Mtb (Figure 5A).

**FIGURE 6:**
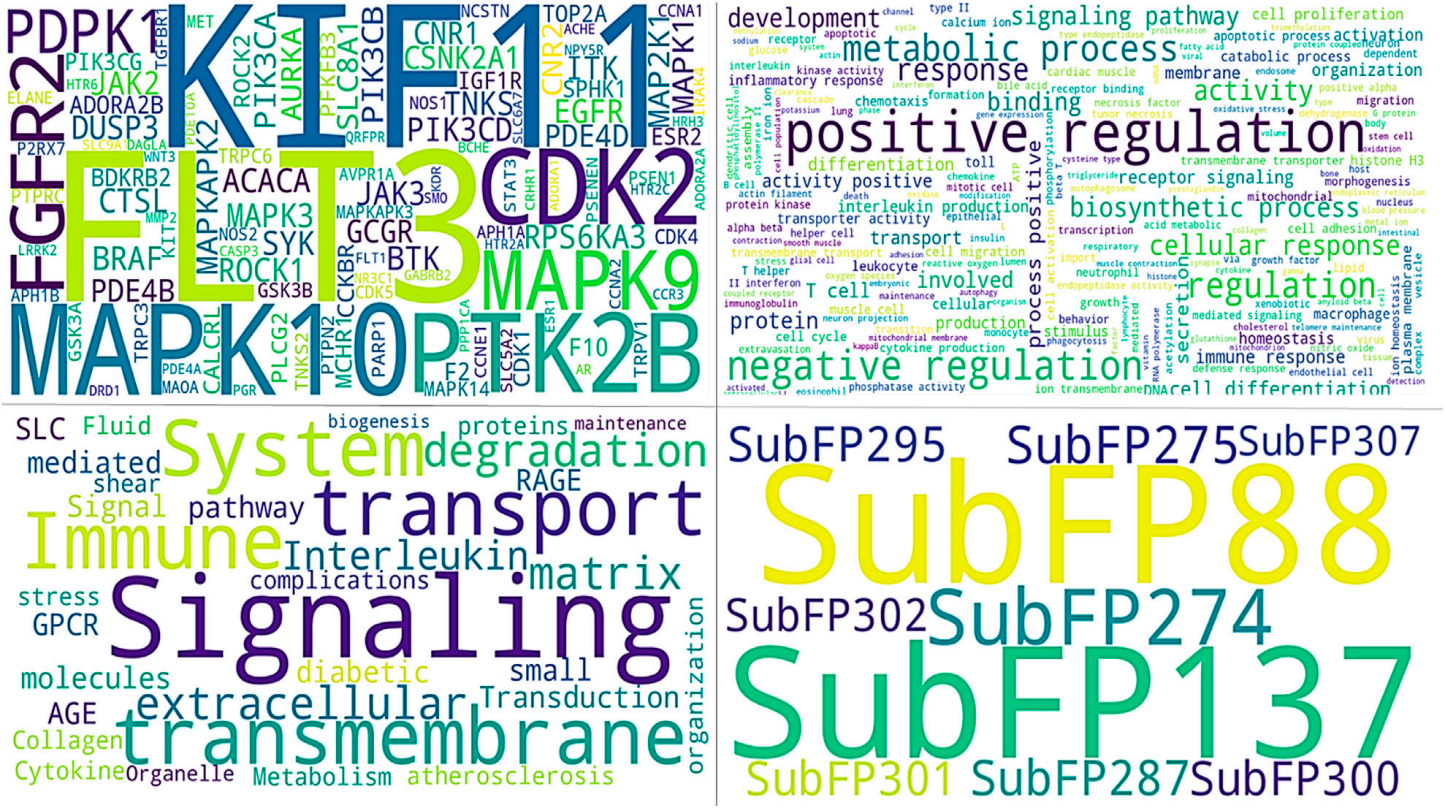
All images show common identified signatures concerning three compounds. The upper panel of the left image shows the genes identified for the top three compounds. The right side of the image displays the names of tuberculosis-associated phenotypes. The lower panel on the right side of the image presents the common Substructure Fingerprint descriptors, while the left image demonstrates the Tuberculosis-associated pathways.

From gene similarity analysis within compounds, we visualized the similarity scores between a list of compounds in heatmap, Venn diagram and word cloud, providing a complete overview of relationships among the compounds (Figure 6). Heatmap of the similarity matrix, demonstrating partial overlap (14.29%) for compound F10921405 compound with F08060425, no overlap (1.82%) between compound F08060425 and F1443-7079, and negligible overlap (1.20%) between compound F10921405 and F14437079. This suggests that F10921405 and F08060425 may share the same mechanisms of action over those shared with F1443-7079. The Venn diagram illustrates the intersecting and non-intersecting genes shared amongst these three molecules. There are 16 genes (13.8% of the total) that have been shared between compounds F10921405 and F08060425 in the analysis. Only one gene is shared between compound F10921405 and F14437079 and between F08060425 and F14437079 (0.9% of total genes analyzed). Importantly, all three compounds had no shared genes and appeared to act through entirely separate pathways or modes of action (Figure 5B). The common genes shared between F10921405 and F0806-0425 include FLT3, MAPK10, CDK2, ITK, SYK, MAPK1, MAPKAPK2, JAK2, PIK3CA, PIK3CB, ROCK1, PDE4B, PDE4D, ADORA2B, PIK3CG, and ROCK2. These genes take part in biological processes like cell signaling, proliferation, and apoptosis, so these two compounds may share similar pathways and have related therapeutic effects. The shared gene between F10921405/F1443–7079 and F08060425/F14437079 does not provide enough evidence to suggest a significant overlap in their mechanisms of action. These compounds, F08060425, F08060425, and F1443-7079, also exhibit extensive overlap revealed by the analysis. These compounds share many common disease-related genes, which is reflected in their high similarity scores. This overlap suggests that these molecules may have similar modes of action against *mycobacterium tuberculosis* (Figure 5B).

Based on phenotype terms, the heatmap demonstrates the similarity matrix based on the (Figure 5D), showing that the similarity percentile between F10921405 and F08060425 is 42.54%, suggesting a moderate level of phenotypic similarity. Indeed, F08060425 has a similarity score of 100% with F1443-7079 (consistent with very high phenotypic similarity between them), potentially meaning that they may share nearly identical or at least very similar phenotypic effects. Lastly, F10921405 has a similarity score of 42.54%, with F14437079 demonstrating moderate phenotypic similarity. This Venn diagram demonstrates the overlap and distinct phenotypic terms that are identified for the three compounds (Figure 4). The analysis reveals that F10921405 and F08060425 share 959 phenotype terms, constituting 57.5% of the total terms analyzed. F10921405 and F14437079, as well as F08060425 and F14437079, share 710 phenotype terms, constituting 42.5% of the total terms analyzed. Notably, all three compounds do not share any common phenotype terms, indicating distinct phenotypic effects for each compound. Image 5C showed mostly similarity percentile between F10921405, F14437079, and F08060425 in the disease state.

From the similarity matrix and Venn diagram analyses, these compounds, F10921405, F08060425, and F14437079, share 15 critical pathways, including the Immune System, SLC-mediated transmembrane transport, Transmembrane transport of small molecules, Signaling by GPCR, and Signal Transduction. The presence of pathways such as AGE-RAGE signaling in diabetic complications and Cytokine Signaling in the Immune System highlights their potential to modulate immune responses, which is crucial for combating TB. Collagen degradation and the Degradation of the extracellular matrix are vital for tissue remodeling during TB infection, affecting granuloma formation and integrity (Squeglia et al., 2018; Whittington et al., 2023). Similarly, pathways like Extracellular matrix organization, Fluid shear stress, and atherosclerosis intersect with immune responses and inflammation, impacting the host’s ability to contain Mtb. Interleukin-4 and 13 signaling, Metabolism of proteins, Signaling by Interleukins, and Organelle biogenesis and maintenance further emphasize the compounds’ roles in regulating immune responses and maintaining cellular function during TB infection. The similarity scores between these compounds provide additional insights. Because the pathway similarity score for Mtb comparison between F10921405 and F08060425 (55.91%) is classified as moderate, this result reinforces that both compounds have significant overlap in their cellular pathways and, therefore, act on similar mechanisms of action against Mtb. On the other hand, the lower similarity score of 27.42% between F10921405 and F14437079 would imply that they target some common and complimentary pathways in addition to unique mechanisms of action. Though the overlap in pathways between F08060425 and F14437079 was limited (similarity 41.38%), an even lower similarity score of 20.45% indicates different MOA, which can be advantageous in combination therapies by providing multiple mechanisms to combat Mtb (Figure 5E). In addition to shared pathways involved in host defences, such as cytokine signaling and immune cell activation, other transmembrane transport pathways are indicated that may enhance the efficacy of host responses against Mtb as targeting or enable anti-TB drugs to reach intracellular targets as modulating. Thus, utilizing common and unique pathways may be an attractive strategy for treating TB in combination therapies that target different aspects of infection pathology.

Based on molecular descriptors provides common descriptors between F10921405 and F08060425, contributing to their high similarity score of 73.79%, include SubFP2, SubFP88, SubFP137, SubFP184, SubFP274, SubFP275, SubFP287, SubFP295, SubFP300, SubFP301, SubFP302, and SubFP307. For the compound F0806–0425 and F1443-7079, with a similarity score of 65.63%, the common descriptors are SubFP88, SubFP137, SubFP274, SubFP275, SubFP287, SubFP295, SubFP300, SubFP301, SubFP302, and SubFP307. Lastly, the common descriptors between F1092–1405 and F1443-7079, contributing to their similarity score of 70.26%, are SubFP88, SubFP137, SubFP274, SubFP275, SubFP287, SubFP295, SubFP300, SubFP301, SubFP302, and SubFP307. Thus, the Venn diagram visually represents the common and unique descriptors among the three compounds. Notably, ten descriptors such as SubFP88, SubFP137, SubFP274, SubFP275, SubFP287, SubFP295, SubFP300, SubFP301, SubFP302, and SubFP307 are common across all compounds, reinforcing their similarity (Figure 5F). The following word cloud illustrates the frequency and importance of these attributes (Figure 6). This relationship between descriptors is important for interpreting the Structure-Activity Relationship (SAR) and gives us a great understanding of how molecular features lead to biological activity. Many of these are shared descriptors and may represent key indicators in the mechanisms of action that underlie the activity of these compounds with biological systems.

### Analysis of gene and pathway frequencies for Mtb targets

**FIGURE 7:**
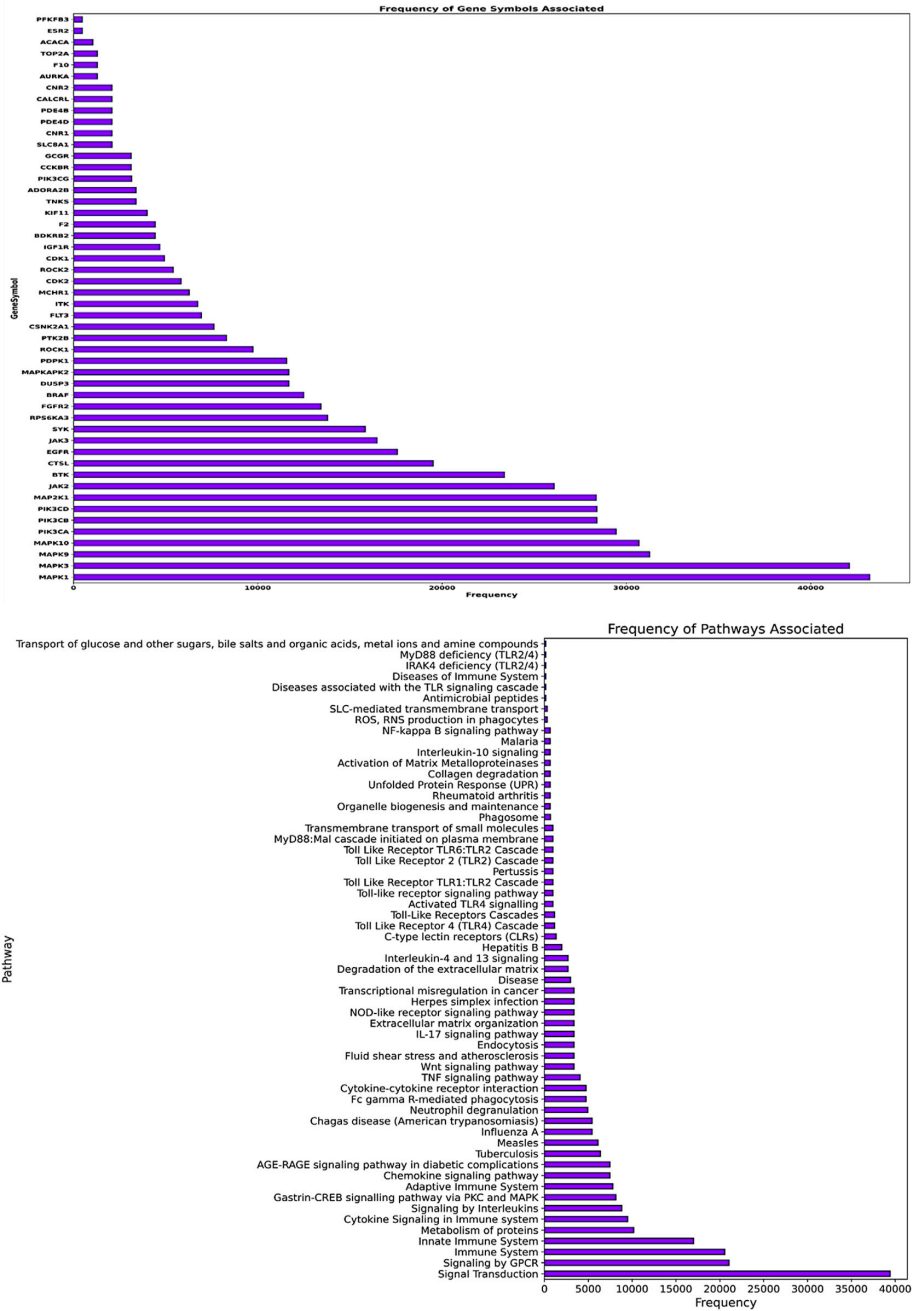
The image’s upper panel shows the frequency of disease-associated genes. The lower panel of the image presents disease-associated pathways.

The gene frequency analysis results of compounds targeting Mtb show interesting associations between their predicted mechanisms based on compound-associated genes (gene symbol). MMP2 has the highest repetition among gene symbols analyzed, suggesting its specificity regarding interaction with these compounds. Matrix Metallopeptidase 2, an extracellular matrix remodeling factor, aids in the degradation of ECM components necessary for tissue remodeling and modulation of the immune response during pathogenesis to Mtb. Other genes such as SMO (Smoothened), HRH3 (Histamine Receptor H3), SLC6A7 (Solute Carrier Family 6 Member 7), CALCRL (Calcitonin Receptor-Like), and NPY5R (Neuropeptide Y Receptor Y5) also exhibit notable frequencies. These genes are involved in signaling transduction, neurotransmitter transport, and cell-to-cell communication, among other biological processes (Figure 7). The involvement of these genes suggests that the compounds may exert their therapeutic effects by modulating multiple pathways essential for Mtb pathogenesis and host-pathogen interactions. Pathway frequency analysis further defines the shared and unique pathways influenced by these Mtb-targeting compounds. The pathway “Immune System” exhibits the highest frequency, underscoring the critical role of immune modulation in combating Mtb infection. This Pathway encompasses various immune responses, including cytokine signaling, antigen presentation, and immune cell activation, vital for mounting an effective defense against the pathogen. Other pathways with high frequencies include “SLC-mediated transmembrane transport,” “Signaling by GPCR,” “Signal Transduction,” “AGE-RAGE signaling pathway in diabetic complications,” and “Cytokine Signaling in the Immune system.” These pathways are crucial for cellular communication, metabolic regulation, and immune responses, strengthening the multi-level defense each of these compounds may offer against Mtb. Pathways such as “Collagen degradation,” “Degradation of the extracellular matrix,” and “Extracellular matrix organization” also show significant frequencies, suggesting that the compounds may affect tissue remodeling and structural integrity, which are essential for controlling Mtb infection and preventing tissue damage (Figure 7).

### Insight from tissue-specific expression and single-cell RNA sequencing analysis

**FIGURE 8:**
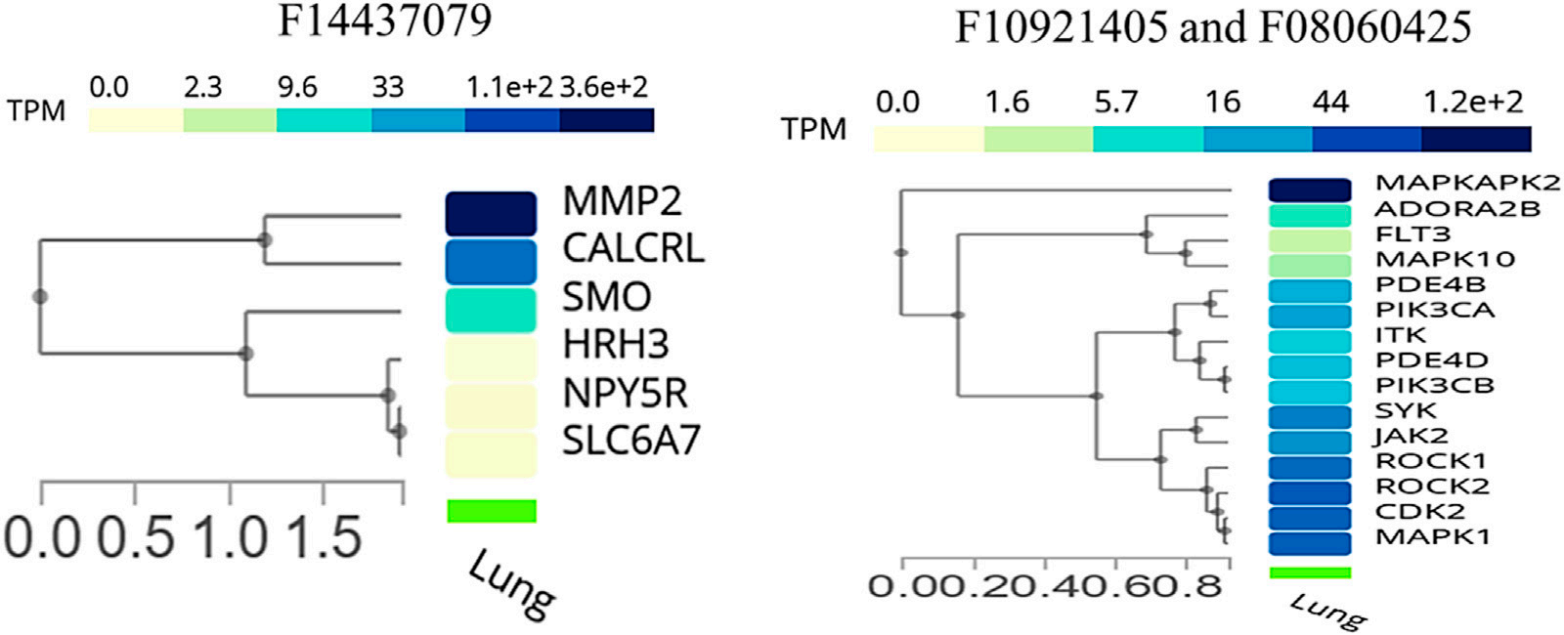
Cluster analysis of tissue-specific expression of a common gene for compound, whereas the right panel of the image shows a common gene between F10921405 and F08060425, and the left panel of the image indicates only single compound F14437079 genes specific expression in lung tissue.

We conducted an expression analysis of the common genes between compounds F1092–1405 and F0806-0425, emphasizing lung tissue, the primary site of infection for Mtb (Figure 8). The heatmap displays visualizes the transcriptional expression levels (measured in Transcripts Per Million, TPM) of selected genes. The heatmap shows that genes exhibit varying expression levels in lung tissue, with TPM values ranging from 0 to over 1.2 * 10^2^. Notably, genes such as MAPKAPK2, MAPK1, and ROCK1 show relatively higher expression levels, indicating their significant presence and potential functional roles in lung tissue. Other genes like ADORA2B and FLT3 are expressed at significantly lower levels, indicating that they likely play a specialized role in specific lung tissues (Figure 8). Analysis of the common genes between compounds F1092–1405 and F0806-0425 in lung tissue expression provides crucial insights into their potential roles in combating Mtb. The higher expression levels of genes like MAPKAPK2, MAPK1, ROCK1, and ROCK2 are particularly noteworthy, as these genes are closely clustered together, suggesting they may have related functions or regulatory mechanisms in the lung tissue response to Mtb. MAPKAPK2 is known for its role in the inflammatory response and cytokine production (Figure 8). Its high expression in lung tissue suggests it may be a pivotal player in modulating the immune response against Mtb. Similarly, MAPK1 (ERK2) gene is part of the MAPK/ERK pathway, which is involved in cell proliferation, differentiation, and response to stress signals. The high expression of MAPK1 gene indicates its potential involvement in the host response to Mtb infection, possibly through the regulation of immune cell functions and inflammatory processes (Peterson et al., 2022).

ROCK1 and ROCK2 are highly expressed genes, playing roles in several cellular processes, including actin cytoskeleton organization cell motility, and represent likely candidates for regulating tissue remodeling and repair after damage induced by Mtb. Its role in the immune response is critical, as it can influence the migration and activation of immune cells, which are necessary for containing and eliminating Mtb (Shi et al., 2013). Moderately expressed genes such as JAK2 and SYK play important roles in cytokine signaling and immune cell regulation. Their levels of expression indicate that these molecules may modulate the host immune responses to Mtb and thereby influence in favor of or against an effective cell-mediated defense (Rojas et al., 2002). The low-to-moderate expression levels of ADORA2B and FLT3 may indicate that the mechanisms associated with these genes might have more localized or transient activity during the implementation of immune responses. ADORA2B mediates its effects by inhibiting pro-inflammatory processes, allowing modulation of immune response and diminishing tissue injury during infection (Ehrentraut et al., 2012). FLT3 is involved in the development and function of immune cells, especially dendritic cells, a key cell type for initiation towards adaptive response against Mtb.

**FIGURE 9:**
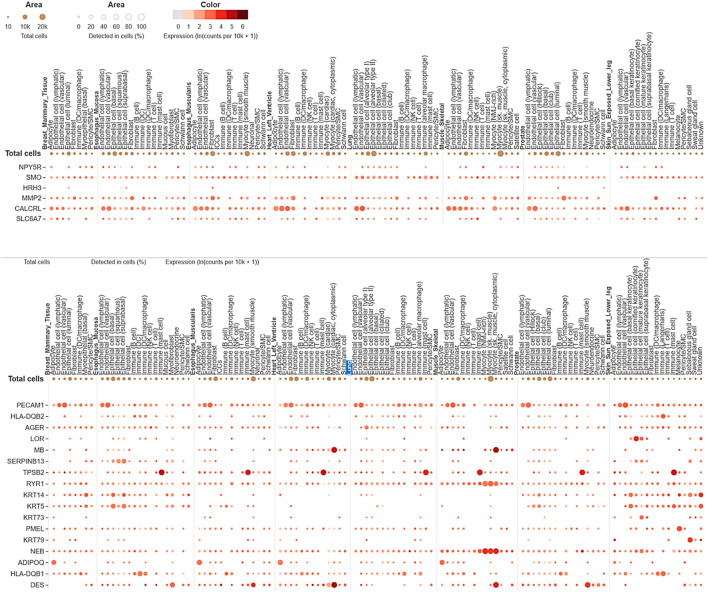
Single-cell RNA sequencing analysis concerning compounds F14437079(Upper image), F10921405, and F08060425 (lower image) in lung-specific tissue and highlights with blue color.

The single-cell RNA sequencing analysis results focused on the expression of common genes between compounds F1092-1405 and F0806-0425 in lung tissue (Figure 9). These genes include PDE4D, MAPK10, and several others, with their expression detected across different cell types within the lung. Specifically, PDE4D expression was detected in 96.78% of epithelial cells (alveolar type II), 77.46% of epithelial cells (basal), 86.61% of epithelial cells (ciliated), 77.88% of epithelial cells (club), 41.07% of immune (B) cells, 38.35% of fibroblasts, and 42.51% of endothelial cells (lymphatic). In alveolar macrophages, PDE4D showed a detection rate of 47.1%, while PDE4B was detected in 47.1%, MAPK10 in 86.38% of ciliated epithelial cells, and 55.26% of alveolar type II epithelial cells. The gene SYK was detected in 44.33% of alveolar macrophages, ROCK1 in 45.11%, and ITK in 41.44% of T cells. The high detection rates of these genes in various lung cell types suggest their significant involvement in lung tissue’s cellular processes and responses. For instance, the wide distribution of PDE4D in various epithelial cells suggests a possible role in preserving their structural integrity and physiological function (Figure 9). Similarly, the presence of MAPK10 in both ciliated and alveolar type II epithelial cells highlights its involvement in essential signaling pathways that could be crucial for lung homeostasis and response to infection. The identification of numerous genes, including SYK, ROCK1, and ITK in immune cells, especially macrophages as well as T cells, further validates the contribution of Mtb infection (Zhang and Liu, 2024).

The expression analysis of MMP2 and CALCRL for compound F1443-7079 showed the significant detection of these proteins in lung endothelial cells, with higher CALCRL detected in vascular endothelial cells (58.08%) than lymphatic endothelial cells (47.01%) (Figure 9). These differential patterns of expression point to a possible role in the vascular and lymphatic systems that may be relevant within the context of Mtb infection and associated inflammatory response. By altering tissue integrity and granuloma formation, MMP2 could profoundly impact lung response to Mtb infection due to its role in extracellular matrix remodeling (Greenlee et al., 2007). This suggests that CALCRL has an important role in regulating vasodilatation and immunity as evidenced by its relatively high level of expression, implying it could be involved in modulating vascular responses and inflammation. SMO and HRH3 are moderately expressed, indicating they may play roles in cell signaling and immune modulation. NPY5R and SLC6A7 are still indicated at lower levels, demonstrating the involvement of neuroimmune pathways that might influence lung defense mechanisms even if their expressions are low (Figure 9). The present findings provide an overview of gene expression in the lungs, emphasizing the variety of roles genes play in different cell types. Their enhanced expression levels within certain cell types could imply a potential contribution to Mtb pathogenesis and host defense mechanisms. The data from this single-cell RNA sequencing analysis contribute to a deeper understanding of lung tissue’s molecular interactions and pathways, potentially guiding targeted therapeutic strategies against Mtb. We conclude that the single-cell RNA-seq analysis is a powerful tool for accurately identifying gene expression patterns across different cell types, allowing for the identification of key genes involved in lung tissue responses to Mtb infection.

## Discussion

The current study sought to accomplish two primary objectives: (i) Homology modeling and virtual screening of antituberculosis compounds against Cas9 protein and (ii) System biology studies in combination with gene expression profiling to investigate the molecular mechanisms associated with *mycobacterium tuberculosis* (Mtb). We started with homology modeling of the Cas9 protein sequence using the high-quality AlphaFold DB model of A0A719XWV6_9MYCO with confidence in its alignment in conserved and active sites. This template was indispensable for creating a high-quality 3D model of the Cas9 protein, on which virtual screening protocols could be based. We curated and filtered compound libraries to identify potential Cas9 inhibitors with antituberculosis activity. Through stringent filtering steps, including drug-likeness and physicochemical filters, toxicophore screening, and PAINS filtration, we refined the libraries to 188 compounds. These compounds underwent energy minimization and molecular docking using PyRx 0.8 with AutoDock Vina, identifying three lead compounds: F10921405, F08060425, and F14437079, which exhibited the highest binding affinities and potential as Cas9 inhibitors. These results indicate a substantial likelihood of these compounds being effective antituberculosis agents.

Moving to our second objective, we developed a computational framework to explore the molecular mechanisms of the identified compounds against Mtb. By integrating multiple data dimensions genes, phenotypes, pathways, and compounds targeting disease-associated genes, we aimed for a comprehensive understanding of tuberculosis. This data was retrieved from the Comparative Toxicogenomic Database. Our analysis revealed that compounds F10921405 and F08060425 shared similar mechanistic insights against Mtb, showing considerable overlap in pathways, genes, phenotypes, and chemical descriptors compared to compound F14437079. The biological networks for compound F10921405 highlights its interactions with critical pathways like chemokine signaling and cytokine signaling in the immune system, involving genes such as MAPK1 and PIK3CA. These interactions suggest that F10921405 could enhance macrophage activation and cytokine production, potentially boosting the immune response against Mtb. Compound F08060425 affects key pathways like cytokine signaling and the Adaptive immune system, interacting with genes FLT3, SYK, and ROCK1, indicating its role in immune modulation and potential applications in cancer therapy. The active compound F14437079 interacts on pathways like IL-4 and 13 signaling, AGE-RAGE signaling, performed genes CDK2, MAPK9, and SMO, proposing its potential to manage diabetology complications and inflammatory conditions. The SM analysis reveals high similarity scores between F08060425 and F10921405 and moderate scores with F14437079, indicating that these compounds could be used synergistically to target multiple aspects of Mtb pathology.

The Similarity Matrix analysis of compounds F08060425, F10921405, and F14437079 provides significant insights into their potential mechanisms of action against Mtb. The percentage of overlapping pathways shared by compounds F08060425 and F10921405 is 49.48%, implying a significant degree of similar mode-of-action between the two compound clusters. F08060425 and F14437079 have a higher similarity score of 73.48%. These results illustrated that they significantly overlapped with each other and thus could probably produce similar therapeutic effects. Besides, the overlapping genes between F10921405 and F08060425 which are implicated in cell signaling, proliferation, or apoptosis, provide a functional link to common therapeutic effects. Compound F14437079 has only a single gene shared by another compound F08060425, suggesting different mechanisms of action. Phenotypic analysis reveals that F10921405 and F0806-0425 share 57.5% of phenotype terms, while F08060425 and F1443-7079 exhibit identical phenotypic effects. The compounds share the common pathways in immune System, SLC-mediated transmembrane transporters, and signaling by GPCR, which underlie their capacities to regulate important immune responses in fighting TB. F10921405 shared a considerable similarity score with F0816F0425 (modulate similar mechanisms), and lower scores with F14437079 which implies unique complementary therapeutic effects.

The single-cell RNA sequencing data reveal the cell-type-specific expression of these genes across various lung cell types, including epithelial cells, immune cells, endothelial cells, and fibroblasts. Compounds F10921405 and F08060425 exhibit similar single-cell RNA sequencing and gene expression profiles, differing from those of compound F14437079. This similarity allows for a better understanding of how these genes function within the diverse cells of the lung, which is crucial in Mtb infection. From the detailed expression profiling, it is clear that genes MMP2, CALCRL, PDE4D, ROCK2, ITK, MAPK10, and SYK have very high levels of expression in specific cell types such as immune cells like epithelial cells, which are vital for the host defense against Mtb. Notably, genes PDE4D, ROCK2, ITK, MAPK10, and SYK are highly expressed in response to compounds F10921405 and F08060425, while genes MMP2 and CALCRL show high expression in response to compound F1443-7079. The high levels of expressions in the immune system, particularly macrophages and T-cells, indicate their possible functions at modulating the immune responses against Mtb infection, while those found in the epithelial cells could be instrumental in maintaining barrier properties to prevent Mtb invasion. A better understanding of heterogeneity within lung tissue would provide insights on how different cell types contribute to overall lung function and its response towards Mtb infection. These expression levels were measured against various types of lung epithelial cells, including type II alveolar cells, basal cells, ciliated cells, mast cells, and club cells. Among the common genes of compounds F10921405 and F08060425, the PDE4D gene was detected in 96.78% of alveolar type II cells, 77.46% of basal cells, 86.61% of ciliated cells, and 77.88% of club cells. The MAPK10 gene was detected in 55.26% of alveolar type II cells, 86.38% of ciliated cells, and 54.09% of club cells. In contrast, for compound F14437079, the expression analysis reveals that MMP2 and CALCRL are significantly detected in lung endothelial cells, with CALCRL showing higher detection in vascular endothelial cells (58.08%) compared to lymphatic endothelial cells (47.01%). The single-cell RNA sequencing analysis data further reveal how these mechanisms and pathways could be targeted in treating Mtb using lung tissue. To study gene expression against Gamma-CA Mtb, further wet lab analysis will be performed to elucidate gene expression analysis and verify the compound’s effectiveness.

## Conclusion

In this study, we explored the biological connections underlying the pathogenesis of *Mycobacterium tuberculosis* (Mtb) and the mechanistic actions of antituberculosis compounds targeting γ-carbonic anhydrase (γ-CA). Through homology modeling and virtual screening, we identified key compounds, F10921405 and F08060425, which may be effective against tuberculosis due to their high binding affinity with the γ-CA protein model. Our systems biology approach integrated genes, phenotypes, pathways, and molecular descriptors, revealing intricate interactions between these compounds and critical biological pathways involved in Mtb pathogenesis. Compounds F10921405 and F08060425 showed significant overlap in their effects on pathways related to immune response, while F1443-7079 displayed distinct mechanistic pathways, emphasizing its role in managing inflammatory conditions. Single-cell RNA sequencing-mediated expression profiling revealed that genes such as PDE4D, ROCK2, ITK, MAPK10, and SYK were regulated in response to F10921405 and F08060425, while MMP2 and CALCRL were regulated in response to F1443-7079. These genes are involved in immune regulation and lung tissue homeostasis, which are critical aspects of fighting Mtb pathogens. We presented the biological relevance of these compounds by elucidating specific molecular determinants and their biological participation in inferring potential efficacy against *Mycobacterium tuberculosis*. Our findings highlight the potential of CA inhibition as a therapeutic strategy for reducing Mtb virulence and advancing tuberculosis treatment. By targeting γ-CA, we aim to disrupt essential metabolic functions and pH balance maintenance in Mtb, providing a novel approach to infectious disease treatment. Future research will focus on further experimental validation of these compounds in clinical settings and explore their efficacy in other infectious disease models.

## Data Availability

The data presented in this study are deposited in the GitHub repository, accessible at https://github.com/Ajay-Manaithiya/Mtb-GammaCA-Mechanism-Cheminformatics.git, and are also available as Supplementary Material accompanying this article.
